# Bracketing Estimation of Intentional Polysubstance Use in the United States

**DOI:** 10.7759/cureus.59653

**Published:** 2024-05-04

**Authors:** Robert B Raffa, Joseph V Pergolizzi Jr.

**Affiliations:** 1 Pharmacology, Temple University, Philadelphia, USA; 2 Pain Management, NEMA Research, Naples, USA

**Keywords:** range, estimation, multiple substance use, polysubstance use disorder, substance use disorder (sud)

## Abstract

Evidence from diverse sources suggests that persons who have a substance use disorder (SUD) often have problems with one or more additional substances, a situation broadly, if imprecisely, termed polysubstance use or more preferably multiple substance use disorder (mSUD). Because of the heavy toll of maladaptive neuronal dysregulation, morbidity, and mortality of SUDs, and increasingly of mSUD, on the individual, their families, the healthcare system, insurers, regulators, and society at large, it seems of value to have an estimate of the prevalence of mSUD. This turns out to be surprisingly difficult, due to nebulous or disparate definitions and to weaknesses in data acquisition methodology. We here attempt a pragmatic way of bracketing an estimate of mSUD prevalence in the US. We conclude that a reasonable estimated range of mSUD in the US is about 8 to 14 million persons. This approach provides a quick estimate for stakeholders involved in efforts to understand or deal with the immediate crisis of mSUD, as more refined estimations are pursued.

## Editorial

In the absence of definitive published data and due to the multiplicity, diverse nature, and variable quality of the available sources, identifying a single number was perceived as unrealistic in the ever-changing real world of substance use. Instead, an attempt was made to ascertain a reasonable range that was consistent with the data and experience and emerged as more useful. In order to try to accomplish this, we applied an approach that would hopefully converge on a range that would be both reasonably large (for inclusivity) and yet small enough to be of practical utility. The goal of this strategy was to try to "bracket" the range for the number of people using multiple drugs of abuse by using existing databases and utilitarian reasoning to estimate: a maximum theoretically possible upper limit, a reasonable minimum possible lower limit, and corrections to yield a smaller range, hopefully reasonable in relation to realistic values by estimated bracketing of the upper and lower values. 

In order to estimate the maximum possible number for multiple substance use disorder (mSUD), we start with the largest individual SUD number. Either alcohol SUD or tobacco use can be used, because the numbers are similar, and furthermore, the estimates for other individual SUD groups (e.g., marijuana, opioids, and stimulants) are much smaller. It should be noted that tobacco use is not universally accepted to be an SUD. However, first, we proffer that it is a substance not used for therapeutic reasons and it is widely known to be deleterious to health; hence, arguendo could qualify as an SUD. Nevertheless, it is inconsequential for the present calculations since the relevant factor is that the prevalence of alcohol SUD and tobacco use is about the same. Further relevant for the present calculations, estimates of the number of tobacco users are likely more reliable compared to other SUD substances because tobacco use is legal above a certain age, there are reliable sales figures, and there is generally less stigma associated with self-report to healthcare providers or in response to a survey. According to the US CDC (Centers for Disease Control and Prevention), the estimated number of adult (≥18 years) cigarette smokers in the US (2021 statistics) is about 28.3 million [1]. The estimation for alcohol SUD is trickier, since the number of people in the US who reported using alcohol on any occasion in the prior month (2021 statistics) was quite high (a "COVID" year), with about 133.1 million total (68.7 million males plus 64.4 million females) but not all of this use qualifies as an SUD. According to the NSDUH (National Survey on Drug Use and Health) by SAMHSA (Substance Abuse and Mental Health Service Administration), the estimated number of heavy alcohol users (≥12 years) in the US (2021 statistics) is about 16.1 million [2]. This number was obtained by defining heavy alcohol use as binge drinking on the same occasion on five or more days in the prior 30 days (binge drinking was defined as drinking five or more drinks by males or four or more drinks by females on the same occasion on at least once in the past 30 days). This seems perhaps a too-stringent definition of an alcohol SUD for our estimate. The NIH NIAA (National Institute on Alcohol Abuse and Alcoholism) estimated (2021 statistics) that 29.5 million people who were 18 years and older in the US had an alcohol SUD [3]. This is a serendipitous number for our estimations since it is very close to the estimated number of cigarette smokers. 

Either the tobacco or alcohol SUD prevalence can be used to establish the upper limit for the prevalence of mSUD due to the fact that they are approximately the same value and far exceed any other individual SUD [2]. Therefore, a combination with one or more other SUD cannot exceed the prevalence of tobacco or alcohol SUD. Hence, based on the estimated prevalence of either tobacco or alcohol SUD, the largest possible prevalence for mSUD is approximately 30 million people. For the actual mSUD to be this number, every tobacco user or person with an alcohol SUD would have to also (mis)use at least one other substance, which is not the case. Thus, 30 million is the hypothetical maximum number. In order to adjust this number to one that is more representative of actual mSUD, we took the two largest SUD groups (tobacco and alcohol) and sought data of overlap. The estimates for the percentage of smokers who also use alcohol vary widely and might be declining: for example, 86% in an oft-cited 1974 publication and 35-45% more recently [4,5]. Therefore, if we use the more recent data, the adjusted high range of mSUD in the US is 10.5-13.5 million people.

Estimation of a lower end for the prevalence range is more challenging since there is no theoretical lower limit (other than the nonsensical 0), inherent methodological weaknesses of estimates of individual SUD prevalence, and the difficulty and nuances of a meaningful and consistent definition of polysubstance use. Therefore, we calculate the prevalence of overlap of each pair of SUDs (since only one overlap is required for mSUD). That is, the intersection of [SUDEtOH] AND {[SUDmarijuana] OR [SUDopioids] OR [SUDstimulants] OR […]}.

SAMHSA has provided an estimate of the intersection of the number of people (≥12 years) in the US (2021 statistics) that have an alcohol SUD (29.5 million in the past year) with those that have a drug-use disorder (27.2 million in the past year). Included in "drug-use disorder" are cocaine, heroin, hallucinogens, inhalants, marijuana, methamphetamine, and prescription psychotherapeutic drugs (i.e., pain relievers, tranquilizers, stimulants, or sedatives). The intersection of alcohol SUD and drug-use disorder is 8 million people [2]. Using this value, the lower limit of mSUD would be about 8 million people (unintentional and inadvertent uses, such as from tainted or "cut" sources, are excluded). 

In conclusion, based on the above approach and approximations and using data provided by US government agencies, it seems that a reasonable range for mSUD in the US is 8-14 million people. In conjunction with other approaches, we hope that this approach contributes to usable estimates and provides additional call-to-action for research, treatment, regulatory, financial, and other valuable purposes.

## References

[1] Cornelius ME, Loretan CG, Jamal A, Davis Lynn BC, Mayer M, Alcantara IC, Neff L (2023). Tobacco product use among adults - United States, 2021. Morb Mortal Wkly Rep.

[2] (2021). National Survey on Drug Use and Health. SAMHSA, Center for Behavioral Health Statistics and Quality. https://www.samhsa.gov/data/data-we-collect/nsduh-national-survey-drug-use-and-health.

[3] (2023). Alcohol and Young Adults Ages 18 to 25. http://NIAAA.Retrievedfromhttps://www.niaaa.nih.gov/alcohols-effects-health/alcohol-topics/alcohol-facts-and-statistics/alcohol-use-united-states-age-groups-and-demographic-characteristics.(2023)..

[4] Friedman GD, Siegelaub AB, Seltzer CC (1974). Cigarettes, alcohol, coffee and peptic ulcer. N Engl J Med.

[5] Anthony JC, Echeagaray-Wagner F (2000). Epidemiologic analysis of alcohol and tobacco use. Alcohol Res Health.

